# The construction and validation of the novel nomograms for the risk prediction of prenatal depression: a cross-sectional study

**DOI:** 10.3389/fpsyt.2024.1478565

**Published:** 2024-11-29

**Authors:** Lanting Huo, Xingfeng Yu, Anum Nisar, Lei Yang, Xiaomei Li

**Affiliations:** ^1^ Faculty of Nursing, Health Science Center, Xi’an Jiaotong University, Xi’an, Shaanxi, China; ^2^ The Nursing Department, Shaanxi Provincial People’s Hospital, Xi’an, Shaanxi, China; ^3^ Institute of Population Health, University of Liverpool, Liverpool, United Kingdom

**Keywords:** prenatal depression, associated factors, prediction model, nomogram, cross-sectional study

## Abstract

**Background:**

Nomograms are superior to traditional multivariate regression models in the competence of quantifying an individual’s personalized risk of having a given condition. To date, no literature has been found to report a quantified risk prediction model for prenatal depression. Therefore, this study was conducted to investigate the prevalence and associated factors of prenatal depression. Moreover, two novel nomograms were constructed for the quantitative risk prediction.

**Methods:**

In this cross-sectional study, the participants were recruited using convenience sampling and administered with the research questionnaires. The prevalence of prenatal depression was calculated with a cutoff point of ≥ 10 in the 8-item Patient Health Questionnaire. Univariate and multivariate binomial logistic regression models were subsequently employed to identify the associated factors of prenatal depression. Two nomograms for the risk prediction were constructed and multiple diagnostic parameters were used to examine their performances.

**Results:**

The prevalence of prenatal depression was 9.5%. Multivariate binomial logistic regression model based on sociodemographic, health-related, and pregnancy-related variables (model I) suggested that unemployment, poor relationship with partners, antecedent history of gynecologic diseases, unplanned pregnancy, an earlier stage of pregnancy, and more severe vomiting symptoms were associated with increased risk of prenatal depression. In the regression model that further included psychosocial indicators (model II), unemployment, antecedent history of gynecologic diseases, unplanned pregnancy, an earlier stage of pregnancy, and a higher total score in the Pregnancy Stress Rating Scale were found to be associated with prenatal depression. The diagnostic parameters suggested that both nomograms for the risk prediction of prenatal depression have satisfactory discriminative and predictive efficiency and clinical utility. The nomogram based on model II tended to have superior performances and a broader estimating range and that based on model I could be advantageous in its ease of use.

**Conclusions:**

The prevalence of prenatal depression was considerably high. Risk factors associated with prenatal depression included unemployment, poor relationship with partners, antecedent history of gynecologic diseases, unplanned pregnancy, an earlier stage of pregnancy, more severe vomiting symptoms, and prenatal stress. The risk prediction model I could be used for fasting screening, while model II could generate more precise risk estimations.

## Background

Pregnancy is a special period, during which women experience dramatic physiological, psychological, and social adaptions. Due to the direct effects of pregnancy and its indirect effects via physiological and social problems, a large proportion of pregnant women are suffering from a variety of psychological disorders (1, 2).

Among the common prenatal psychological disorders, depression is the most remarkable, considering its high prevalence and heavy health burdens. A systematic review involving 197,047 participants showed a pooled prenatal depression prevalence of 20.7% (3). Such figure is 19.7% among Chinese pregnant women as reported in a recent systematic review with meta-analysis (4). Prenatal depression and depressive symptoms could lead to a large spectrum of adverse birth outcomes, such as preeclampsia, gestational diabetes, slow fetal growth, low birth weight, preterm birth, miscarriage, stillbirth, and impaired mother-baby bonding (5–8). In addition, the long-term consequences of prenatal depression were also frequently reported in the literature, which included long-lasting mental problems in mothers, and poor growth and development in offsprings (9, 10).

Despite its high prevalence and far-reaching negative consequences, the attention paid to prenatal depression is far from enough. On the one hand, regular screening of the condition is missing in the clinical setting. A recent population-based investigation suggested that the prevalence of undiagnosed prenatal depression was up to 82.3% (11). On the other hand, a diagnosis of prenatal depression could be mistakenly precluded due to the shared symptoms of depression and normal pregnancy, for example, fatigue and appetite loss (12). Moreover, pregnant women could be hesitant to report depressive symptoms, which are viewed as stigmas in some cultures (13). Thus, the early detection of prenatal depression is elementary for the management of the condition and the improvement of the well-being of pregnant women and their offspring.

The construction of risk prediction models based on selected risk factors associated with prenatal depression is beneficial for the recognition of vulnerable groups and the implementation of early preventive and management strategies. Existing evidence has suggested some associated factors of prenatal depression, which included age, socioeconomic status, social support, antecedent history of physiological and psychological illness, unplanned pregnancy, stage of pregnancy (trimester), number of gestations, and pregnancy complications (3, 4, 14). Although some of these studies have examined the association between the abovementioned variables and prenatal depression using multivariate regression models, their clinical significance could be limited (15). For one thing, the association between some variables and prenatal depression remains inconclusive. For example, some studies reported an increased risk of having prenatal depression with the progression of pregnancy, while other studies suggest a tendency of gradually decreased risk (3, 16, 17). For another thing, some important factors are neglected in the existing models, such as perceived relationship with mother-in-law [a substantial source of social support for Chinese pregnant women as nearly one-third of them live with their mothers-in-law (18)], the severity of vomiting symptoms (19, 20), and the antecedent history of gynecologic diseases (20, 21). More importantly, admitted that multivariate regression models can help identify the associated factors of given conditions, they cannot help quantify an individual’s risk of having the conditions.

Nomogram, a recently proposed risk prediction tool, meets the need for individualized risk quantification and has been widely used for risk prediction in the medical field (22, 23). The nomogram is a graphical tool to present the results of a multivariate regression model that predicts the probability of a given event by integrating the relative contributions of each risk factor in the predictive model. This allows the risk value of each factor to be quantified according to the proportion and thus enables personalized risk quantification (24). A nomogram was developed for the risk prediction of prenatal depression recently (23). However, despite the large sample size, it is also criticized for neglecting some substantial risk factors, for example, the stage of pregnancy.

To address the abovementioned research gaps, the current study was conceptualized with the objective of investigating the prevalence and associated factors of prenatal depression. Moreover, two novel nomograms were constructed for the quantitative risk prediction of prenatal depression.

## Materials and methods

The reporting of this study adhered to the Strengthening the Reporting of Observational Studies in Epidemiology (STROBE) statement (25).

### Study design, setting and participants

This study employed a cross-sectional study design. The field survey was carried out in two university-affiliated, governmental, tertiary hospitals in Xi’an City, Northwestern China, from July to August 2020.

The target population was the pregnant women who visited the obstetrics clinic of the two research sites for routine prenatal health assessments. The inclusion criteria for the research participants included: 1) aged ≥ 18 years; 2) cognitively independent; 3) with sufficient communication ability; 4) consent to participation. Individuals were excluded if they were diagnosed with any mental disorder or were participating in other studies. The research participants were recruited on a convenient basis, with all eligible pregnant women approached for participation invitation during the study period.

We did not perform formal sample size calculation because there are no generally acknowledged approaches to sample size estimation for studies on the construction and validation of prediction models (26). Because this is a secondary analysis based on the available dataset of a prevalence cross-sectional study, it makes sense to use the entire dataset, per guidelines from Moons (26).

### Variables and measurements

The variables measured in this study included the outcome variable, namely prenatal depression, and the input variables, referring to the potential associated factors of the condition. The input variables were prespecified based on a comprehensive review of evidence regarding the associated factors of prenatal depression, which include a variety of sociodemographic, health-related, and pregnancy-related variables and several psychosocial variables. Exceptionally, anxiety was not included in the analysis of this study even though the condition was reported to be associated with prenatal depression (4). This decision was made under the consideration that anxiety and depression have shared underlying pathophysiological mechanisms, risk factors, and somatic symptoms, making it difficult to discriminate between the two conditions (27).

#### Sociodemographic, health-related and pregnancy-related variables

Sociodemographic, health-related, and pregnancy-related variables were collected with a researcher-designed information sheet. The investigated variables included age, education level, employment status, monthly household income, residency, relationship with the partner, relationship with mother-in-law, pre-pregnancy body mass index (BMI), history of gynecological diseases, history of dysmenorrhea, stage of pregnancy, gravity, unplanned pregnancy, the severity of vomiting symptoms, abnormal pregnancy indicators, and monocyesis.

#### Prenatal depression

Prenatal depression was assessed using the 8-item Patient Health Questionnaire (PHQ-8). The PHQ-8 is a shortened version of the PHQ-9, which has been widely used to assess depression and demonstrated good psychometric properties among Chinese pregnant women (28). The PHQ-8 encompasses eight of the nine diagnostic criteria for major depression as mentioned in the Diagnostic and Statistical Manual of Mental Disorders - 5th Edition (DSM-5), with the question about suicidal and/or self-harm ideation deleted (29, 30). The instrument assesses the informants’ depressive symptoms during the past two weeks. Each item was scored from 0 (not at all) to 3 (nearly every day) and the possible range for the total score is 0−24, with higher scores indicating more severe depressive symptoms. A cutoff point of ≥ 10 was suggested to define clinical/current depression. The PHQ-8 is equivalent to the PHQ-9 and has good reliability and validity among pregnant women (31, 32). The Cronbach’s alpha coefficient in the current study was 0.81.

#### Pregnancy stress

Pregnancy stress was assessed employing the 30-item Pregnancy Stress Rating Scale (PSRS), developed by Chen and colleagues in 1983 (33). It consists of three dimensions, which are stress of maternal and infant health and safety, stress of maternal role identification, and stress of altered physical appearance and function. Items were rated on a 4-point Likert scale ranging from 0 (definitely no) to 3 (very severe), which could generate a total score of 0−90. Higher scores represent higher levels of perceived stress. The original Chinese version of the PSRS had good psychometric properties (34). In the current study, the Cronbach’s alpha coefficients for different dimensions and the total scale were between 0.74 to 0.93.

#### Perceived social support

Social support perceived during pregnancy was assessed with the Multidimensional Scale of Perceived Social Support (MSPSS), developed by Zimet and colleagues in 1988 (35). The MSPSS assesses an individual’s subjective perception of social support from three dimensions, including family, friends, and others, with each dimension comprising four items. Items were rated on a 7-point Likert scale ranging from 1 (very strongly disagree) to 7 (very strongly agree), and a higher score indicated a higher level of perceived social support. The instrument has exhibited outstanding psychometric properties in multiple populations (36). In this study, Cronbach’s alpha coefficient for the full scale and different dimensions was between 0.79 to 0.85.

#### Coping style

Coping style was assessed employing the Simplified Coping Style Questionnaire (SCSQ), a self-rating instrument developed by Xie in 1998 (37). The 20 items in the instrument could generate two dimensions, defined as “active coping (12 items)” and “passive coping (8 items)”. Items were rated on a 4-point Likert scale ranging from 0 (never) to 3 (always), and the possible scores for the active coping dimension and the passive coping dimension were 0−36 and 0−24, respectively. A high score on each dimension indicates a more frequent usage of that coping style. The SCSQ exhibited good reliability and validity in different populations (37, 38). The Cronbach’s alpha coefficient for the total scale and two subscales was greater than 0.75 in this study.

### Procedures

Upon the identification of a potentially eligible participant, the principal investigator elaborated the objectives, procedure, and significance of the study, and invited the pregnant woman to participate. Interested participants were required to provide written informed consent and, subsequently, administered with the research questionnaires. In a face-to-face manner, the principal investigator asked the questions one by one following the protocolized procedure and recorded the participants’ responses in the printed survey questionnaires. All data collection was conducted by the principal investigator, and thus, consistency across the collected data was maximized.

### Ethical considerations

This study obtained ethical approval from the Ethics Committee of the Health Science Center, Xi’an Jiaotong University (reference identifier: 2020-1373) and permissions from the participating hospitals. Written informed consent was obtained from the participants before data collection. The participants’ rights and safety were protected by adhering to national and local laws/regulations, the Declaration of Helsinki, and institutional policies. Participants who scored ≥ 10 points in the PHQ-8 were referred to the psychological clinics of a local tertiary hospital for professional assessments and assistance on a willing basis.

### Statistical analysis

The IBM SPSS version 22.0 and R version 4.4.0 were used for data analysis. Continuous data with normal distribution were presented as mean and standard deviation, while categorical data as count and percentage. Using the classical split-sample validation approach, the total samples were randomly assigned to the training set and the validation set at a ratio of 7:3 (26), serving as risk model (nomogram) construction and validation, respectively. The homogeneity in the sociodemographic, health-related, and pregnancy-related characteristics between sets was tested using the independent t-test, Chi-square test, or Fisher’s exact test where appropriate.

Univariate logistic regression models were employed to screen the candidate factors associated with prenatal depression, with a statistical significance level of α=0.1 (39). Subsequently, the candidate factors were entered into the multivariate binomial logistic regression models to identify the independent associated factors of prenatal depression, with a statistical significance level of α=0.05. Based on the results of multivariate binomial logistic regression models, two nomograms for the risk prediction of prenatal depression were constructed using the R package “rms”. The associated factors were ordered based on their importance using the mean decrease Gini method. Multiple parameters were employed to examine the performance of the novel prediction models. The receiver operating characteristic (ROC) curve with area under the curve (AUC) and the net classification improvement (NRI) were utilized to assess the discriminative efficiency of the prediction models. The calibration curves, which demonstrate the differences between the predicted and observed probability, the Hosmer-Lemeshow test with a 1,000-sample bootstrap, and the integrated discrimination improvement (IDI) were employed to examine the predictive efficiency of the prediction models. The decision curve analysis (DCA), which quantifies the net benefits at different threshold probabilities, was used to evaluate the clinical utility of the prediction models.

## Results

### Sociodemographic, health-related, pregnancy-related and psychosocial characteristics of the research participants

A total of 1,465 pregnant women were invited to participate, of which 1,020 (69.62%) agreed and 999 (68.19%) provided complete data. The age of the participants ranged from 21 to 44 years, with an average of 30.12 (standard deviation: 3.46). Around one-fifth and 50% of the participants had a history of gynecologic diseases and dysmenorrhea, respectively. More than half of the participants were in the third trimester and two-fifths had an unplanned pregnancy.

The participants averaged a total score of 45.88 (standard deviation: 11.97) and 60.61 (standard deviation: 13.37) in the PSRS and MSPSS, respectively. For the SCSQ, the participants had a mean score of 24.58 (standard deviation: 5.72) in the active coping dimension and 9.91 (standard deviation: 4.35) in the passive coping dimension.

Participants in the training and validation sets were homogeneous in all sociodemographic, health-related, pregnancy-related, and psychosocial characteristics. Detailed characteristics of the research participants are presented in **Table 1**.

**Table 1:** Characteristics of the total samples and the compassion between the training and validation sets (N=999).

Variables	Total samples (N=999)	Training set (n=693)	Validation set (n=306)	Test statistics, *P* value
**Age [in years, Mean** ± **SD]**	30.12 ± 3.46	30.06 ± 3.36	30.25 ± 3.68	t=-0.798, 0.425
**Marital duration [in years, Mean ± SD]**	3.60 ± 3.08	3.54 ± 3.13	3.74 ± 3.42	t=-0.843, 0.400
Pre-pregnancy BMI				
< 18.5	162(16.38)	115(16.69)	47(15.67)	χ^2^ = 1.914, 0.590
18.5-23.9	674(68.15)	471(68.36)	203(67.66)	
24.0-27.9	127(12.84)	83(12.05)	44(14.67)	
≥ 28	26(2.63)	20(2.90)	6(2.00)	
Educational level				
High school degree or lower	112(11.28)	78(11.30)	34(11.22)	χ^2^ = 4.721, 0.094
College degree	279(28.10)	180(26.09)	99(32.67)	
Undergraduate degree or above	602(60.62)	432(62.61)	170(56.11)	
Employment status				
Housewife	194(22.0)	133(21.70)	61(22.85)	χ^2^ = 0.143, 0.705
Employed	686(78.0)	480(78.30)	206(77.15)	
Relationship with partner				
Close	739(78.8)	505(77.57)	234(81.53)	χ^2^ = 1.894, 0.388
Good	174(18.6)	128(19.66)	46(16.03)	
General	25(2.6)	18(2.77)	7(2.44)	
Relationship with mother-in-law				
Close	487(53.4)	335(53.17)	152(53.71)	χ^2^ = 0.023, 0.988
Good	276(30.2)	191(30.32)	85(30.04)	
General	150(16.4)	104(16.51)	46(16.25)	
Living alone				
Yes	25(2.7)	20(3.07)	5(1.75)	χ^2^ = 1.317, 0.251
No	912(97.3)	632(96.93)	280(98.25)	
History of gynecologic disease				
Yes	176(19.3)	121(19.15)	55(19.50)	χ^2^ = 0.016, 0.899
No	738(80.7)	511(80.85)	227(80.5)	
History of dysmenorrhea				
Yes	443(47.8)	304(47.43)	139(48.77)	χ^2^ = 0.143, 0.705
No	483(52.2)	337(52.57)	146(51.23)	
Trimester (stage of pregnancy)				
< 3month	152(15.3)	99(14.37)	53(17.55)	χ^2^ = 1.696, 0.428
3-6 month	298(30.1)	208(30.19)	90(29.80)	
> 6month	541(54.6)	382(55.44)	159(52.65)	
Gravity				
Primigravida	607(66.6)	427(61.62)	180(58.82)	χ^2^ = 0.694, 0.405
Multigravida	304(30.4)	266(38.38)	126(41.18)	
Unplanned pregnancy				
Yes	425(42.8)	293(42.40)	132(43.85)	χ^2^ = 0.180, 0.671
No	567(57.2)	398(57.60)	169(56.15)	
Abnormal pregnancy indicators				
Yes	24(2.4)	18(2.64)	6(1.98)	χ^2^ = 0.379, 0.538
No	962(97.6)	665(97.36)	297(98.02)	
Severity of vomiting symptoms				
No or minimal	235(23.6)	162(23.45)	73(24.01)	χ^2^ = 0.059, 0.971
Mild	349(35.1)	242(35.02)	107(35.20)	
Moderate to severe	411(41.3)	287(41.53)	124(40.79)	
Baby gender expectation				
Boy	123(12.3)	81(11.69)	42(13.82)	χ^2^ = 0.889, 0.641
Girl	255(25.6)	179(25.83)	76(25.00)	
No	619(62.1)	433(62.48)	186(61.18)	
**Total score in the PSRS [Mean ± SD]**	45.88 ± 11.97	45.41 ± 11.64	46.96 ± 12.67	t=-1.790, 0.074
**Total score in the MSPSS [Mean ± SD]**	60.61 ± 13.37	60.51 ± 13.83	60.83 ± 12.29	t=-0.348, 0.728
**Active coping dimensional score in the SCSQ [Mean ± SD]**	24.58 ± 5.72	24.79 ± 5.81	24.12 ± 5.52	t=-1.594, 0.111
**Passive coping dimensional score in the SCSQ [Mean ± SD]**	9.91 ± 4.35	10.00 ± 4.50	9.73 ± 4.00	t=-0.854, 0.393
**Total score in the PHQ-8 [Mean ± SD]**	5.65 ± 3.66	5.59 ± 3.65	5.77 ± 3.69	t=-0.71, 0.477
< 10	869(90.5)	605(90.7)	264(90.1)	χ^2^ = 0.086, 0.769
≥ 10	91(9.5)	62(9.3)	29(90.9)	

PHQ-8, 8-item Patient Health Questionnaire; PSRS, Pregnancy Stress Rating Scale; MSPSS, Multidimensional Scale of Perceived Social Support; SCSQ, Simplified Coping Style Questionnaire.

Bold text indicates variable names.

### Prevalence and associated factors of prenatal depression

The participants had an average of 5.65 (standard deviation: 3.66) in the PHQ-8 total score. According to a cut-off point of ≥ 10, the prevalence of prenatal depression in the current study was 9.5% (**Table 1**).

Multivariate binomial logistic regression model based on sociodemographic, health-related, and pregnancy-related variables (model I) suggested that unemployment, poor relationship with partners, the antecedent history of gynecologic diseases, unplanned pregnancy, an earlier stage of pregnancy, and more severe vomiting symptoms were associated with increased risk of prenatal depression (**Table 2**). In the regression model that further included psychosocial indicators (model II), unemployment, the antecedent history of gynecologic diseases, unplanned pregnancy, an earlier stage of pregnancy, and a higher total score in the PSRS were found to be associated with prenatal depression (**Table 2**).

**Table 2:** Univariate and multivariate analysis of factors associated with prenatal depression (N=693).

Variables	Univariate analysis		Multivariate analysis			
			Model I		Model II	
	OR	95%CI	OR	95%CI	OR	95%CI
**Age**	0.992	0.931 to 1.057				
**Marriage duration**	1.020	0.954 to 1.091				
Employment status						
Housewife	1	Reference	**1**	**Reference**	**1**	**Reference**
Employed	0.554^**^	0.339 to 0.904	0.552**	**0.318 to 0.957**	0.536**	**0.288 to 0.995**
Living alone						
No	1	Reference	1	Reference	1	Reference
Yes	3.182^**^	1.235 to 8.199	2.803	0.935 to 8.406	1.737	0.422 to 7.155
Relationship with partner						
General	1	Reference	**1**	**Reference**	1	Reference
Good	0.680	0.325 to 1.426	**0.417**	**0.136 to 1.279**	0.618	0.146 to 2.625
Close	0.437**	0.232 to 0.824	0.249**	**0.081 to 0.768**	0.359	0.082 to 1.564
Relationship with mother-in-law						
General	1	Reference	1	Reference	1	Reference
Good	0.687	0.387 to 1.217	0.775	0.380 to 1.577	1.040	0.437 to 2.475
Close	0.6128*	0.367 to 1.021	0.859	0.401 to 1.842	1.482	0.565 to 3.884
History of gynecologic diseases						
No	1	Reference	**1**	**Reference**	**1**	**Reference**
Yes	1.916^**^	1.160 to 3.166	1.786**	**1.024 to 3.115**	1.879**	**1.004to 3.514**
History of dysmenorrhea						
No	1	Reference				
Yes	1.334	0.856 to 2.078				
Gravity						
Primigravida	1	Reference				
Multigravida	1.192	0.742 to 1.913				
Unplanned pregnancy						
No	1	Reference	**1**	**Reference**	**1**	**Reference**
Yes	2.040^**^	1.312 to 3.171	2.206**	**1.328 to 3.665**	2.381**	**1.319 to 4.301**
Abnormal pregnancy indicators						
Yes	1	Reference				
No	1.922	0.642 to 5.750				
Trimester (stage of pregnancy)						
<3month	1	Reference	**1**	**Reference**	**1**	**Reference**
3-6 month	0.407^**^	0.234 to 0.706	0.307**	**0.162 to 0.581**	0.301**	**0.144 to 0.629**
>6month	0.237^**^	0.139 to 0.403	0.205**	**0.112 to 0.376**	0.205**	**0.100 to 0.417**
Severity of vomiting symptoms						
No or minimal	1	Reference	**1**	**Reference**	1	Reference
Mild	1.854*	0.931 to 3.692	**1.733**	**0.808 to 3.714**	1.973	0.829 to 4.695
Moderate to severe	2.483^**^	1.290 to 4.779	2.257**	**1.100 to 4.630**	2.159	0.947 to 4.921
Baby gender expectation						
No	1	Reference	1	Reference	1	Reference
Boy	1.419	0.743 to 2.710	1.429	0.685 to 2.980	1.470	0.629 to 3.435
Girl	1.606*	0.991 to 2.603	1.295	0.738 to 2.274	1.547	0.812 to 2.948
**Total score in the PSRS**	1.065**	1.046 to 1.082	–	–	5.477**	**2.864 to 10.476**
**Total score in the MSPSS**	0.976**	0.960 to 0.992	–	–	0.869	0.659 to 1.146
**Active coping dimensional score in the SCSQ**	0.956*	0.922 to 0.992	–	–	0.874	0.434 to 1.758
**Passive coping dimensional score in the SCSQ**	1.050*	0.997 to 1.106	–	–	1.404	0.778 to 2.534

PSRS, Pregnancy Stress Rating Scale; MSPSS, Multidimensional Scale of Perceived Social Support; SCSQ: Simplified Coping Style Questionnaire; *p < 0.1(for univariate analysis only); **p<0.05.

Bold text indicates variable names. Bold figures indicate statistical significance (**p<0.05).

### Construction and validation of the nomograms for the risk prediction of prenatal depression

**Figure 1:**
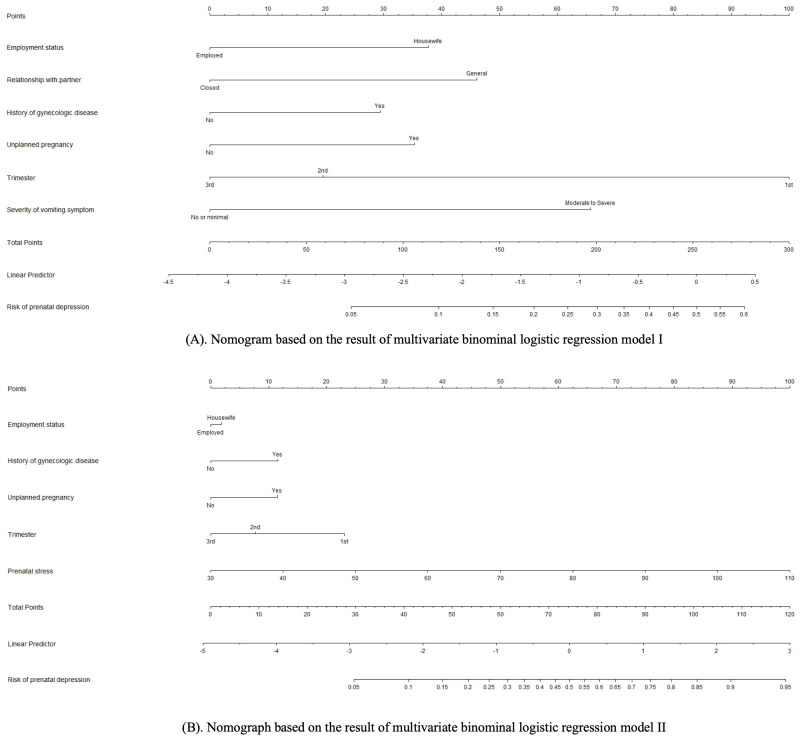
**(A)** Nomogram based on the result of multivariate binominal logistic regression model I. **(B)** Nomograph based on the result of multivariate binominal logistic regression model II.

Based on the multivariate binominal logistic regression models, two individualized nomogram models for the risk prediction of prenatal depression were constructed (**Figure 1**), with the input variables ranked in a descending manner based on their importance (**Supplementary Figure 1**). The nomograms assigned a specific score to each input variable, and the total score was calculated as the sum of these individual scores. The predicted risk of prenatal depression was thereafter determined based on the corresponding probability associated with the total score.

**Figure 2:**
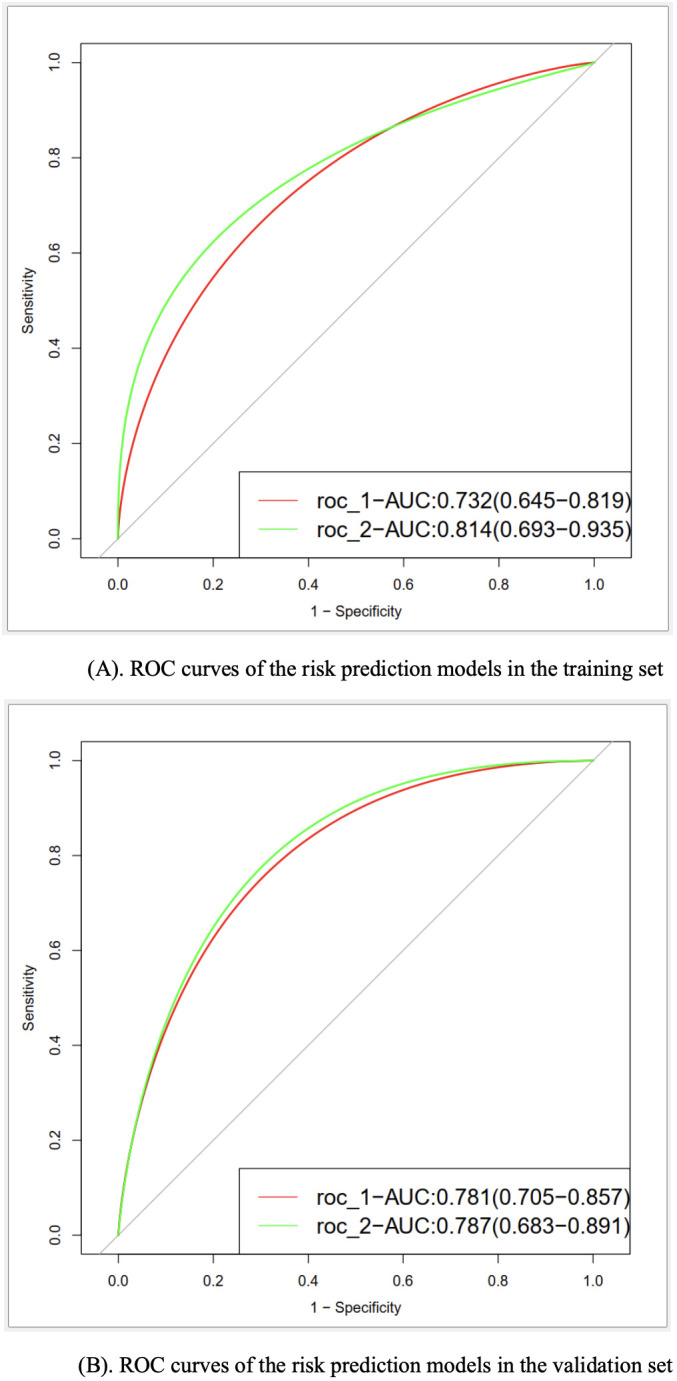
**(A)** ROC curves of the risk prediction models in the training set. **(B)** ROC curves of the risk prediction models in the validation set.

In the training set, the AUC values for model I and model II were 0.732 and 0.814, respectively. Meanwhile, the values were 0.781 and 0.787 in the validation set, respectively (**Figure 2**). Such results indicated that both models had good discriminative efficiency, but model II was superior compared to model I. Furthermore, model II was found to have higher reclassification efficiency (NRI: 0.645 [95% CI, 0.314−0.981]; *P*<0.001) compared to model I, which supported the superior discriminative performance of the former.

**Figure 3:**
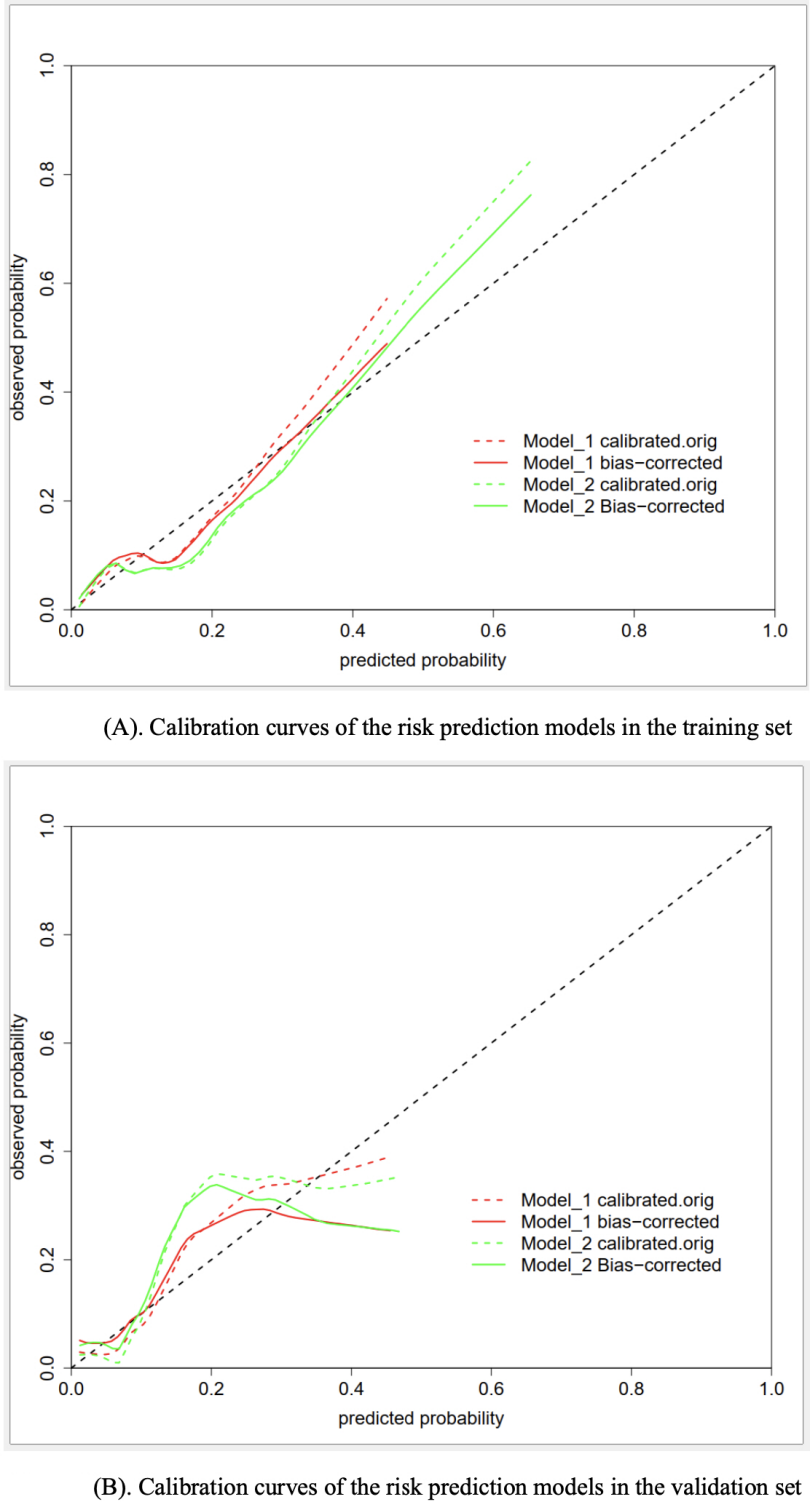
**(A)** Calibration curves of the risk prediction models in the training set. **(B)** Calibration curves of the risk prediction models in the validation set.

Concerning the predictive performance, the calibration curves demonstrated a moderate level of agreement or consistency between the predicted risk and actual observation (**Figure 3**). Besides, the coefficients of the Homser-Lemeshow test for model I and model II were 11.473 (*P*=0.1781) and 11.979 (*P*=0.1521), respectively, which supported the good predictive efficiency of both models. However, the IDI coefficient (0.120, 95% CI: 0.068−0.173; *P*<0.001) suggested that model II tended to have improved predictive efficiency compared to model I.

**Figure 4:**
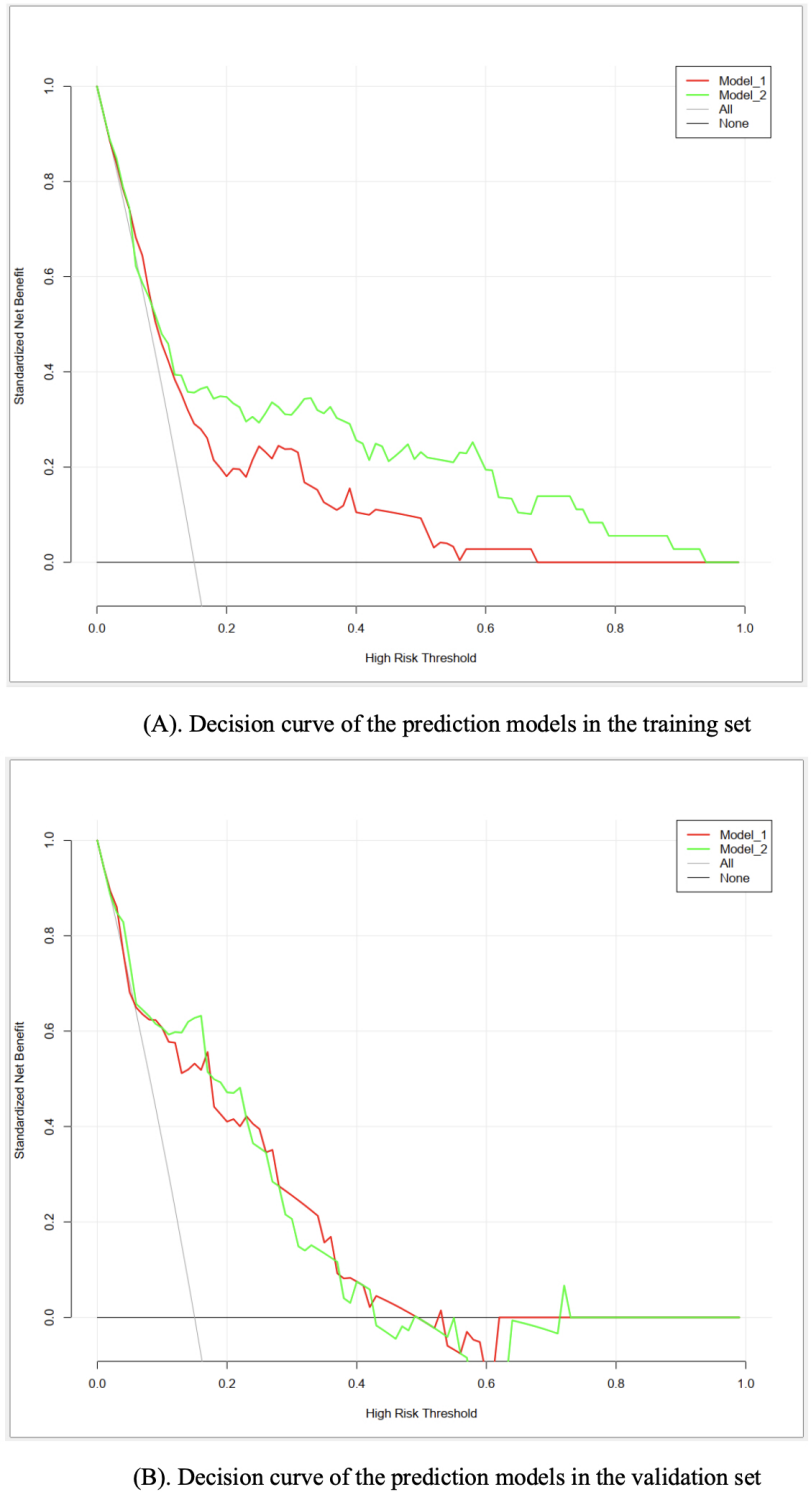
**(A)** Decision curve of the prediction models in the training set. **(B)** Decision curve of the prediction models in the validation set.

As for the clinical utility, the results of DCA suggested that both models had significant net benefits in most of the probability thresholds (**Figure 4**), indicating good performance in guiding clinical decisions. By contrast, model II exhibited higher net benefits in the probability threshold of 0.2−0.95, compared to model I.

## Discussion

Prenatal depression is a common psychological disorder that imposes multidimensional burdens on pregnant women and their offspring. Identifying individuals with high vulnerability to the condition is elementary for the implementation of early preventive and management strategies. The results of this study showed a considerably high prevalence of prenatal depression in pregnant women. Two nomograms for the risk prediction of prenatal depression were constructed based on the results of multivariate binominal logistic regression models which included multiple sociodemographic, health-related, pregnancy-related, and psychosocial risk factors. Both nomograms were found to have good discriminative and predictive performance and clinical utility. The nomogram based on model II tended to have superior performances and a broader estimating range, while by contrast, the nomogram based on model I could be advantageous in its ease of use.

### Prenatal depression was common in pregnant women

The prevalence of prenatal depression in the current study was 9.5%, which is significantly lower than the reported figures of approximately 20% in recent systematic reviews with meta-analyses (3, 4). The major reason for the significantly lower prevalence of prenatal depression in the current study could be its strict operational definition of depression. A cutoff point of ≥ 10 was adopted, which represents major depression or clinically significant depression (29, 40). Such a hypothesis is partly supported by a large-scale investigation that reported a prevalence of 6.1% for prenatal depression using the same criterion (40). Another explanation for the low prevalence of prenatal depression in our study might be the fact that a dominant proportion of the samples had a college degree or above, as a higher educational level has been viewed as a protective factor of the condition (4).

### Multiple sociodemographic, health-related, pregnancy-related and psychosocial factors were associated with prenatal depression

The result of this study showed that employed women were less likely to experience prenatal depression compared to unemployed women or housewives, with an odds ratio (OR) of 0.55 in model I and 0.54 in model II. Existing evidence tended to be consistent with the current study in supporting an association between unemployment and increased risk of prenatal depression (3, 41). Some researchers believe that unemployed women have a higher risk of prenatal anxiety and stress, which are known contributors to prenatal depression (42). Besides, unemployment might also lead to heavier economic burdens, more family conflicts, and less social interaction, which could further aggravate prenatal depression (39, 43). Participants rated a close relationship with their partner had a lower risk of prenatal depression compared to those rated a general relationship (OR: 0.25 in model I), which is consistent with the findings of previous studies (14, 44). The husband is the most important source of affective value and social support for pregnant women, and thus, a poor spousal relationship would inevitably increase the risk of prenatal depression.

It was found in this study that pregnant women who had an antecedent history of gynecologic diseases were more vulnerable to prenatal depression (OR: 1.79 in model I and 1.88 in model II). Even though relevant evidence is lacking, pregnant women may probably worry that the antecedent gynecologic diseases could result in adverse pregnancy outcomes, which could further lead to psychological burdens. Anyhow, considering the lack of direct evidence on the association between the antecedent history of gynecologic diseases and prenatal depression, a conclusion cannot be drawn and further investigations are valuable.

Accordant with existing evidence, unplanned pregnancy was found to increase the risk of prenatal depression in this study (OR: 2.21 in model I and 2.38 in model II) (3, 45, 46). Human beings find it more difficult to cope with unexpected and undesired events (47). Similarly, women could be more likely to experience psychological problems in the face of an unplanned pregnancy. Compared to those in the first trimester, participants in the second and third trimester were less likely to have depression in this study. Even though subgroup analysis of a systematic review suggested that the pooled prevalence of prenatal depression was the highest in studies that recruited pregnant women in the third trimester (3), updated longitudinal and large-scale multicenter cross-sectional evidence has supported the findings of the current study (16, 17). To further clarify the relationship between the stage of pregnancy and prenatal depression, more empirical studies are encouraged. The decreasing tendency in the risk of prenatal depression during pregnancy could be interpreted as the result of a gradual adaption to the stressful event of pregnancy. The results of multivariate binomial logistic regression for model I indicated that pregnant women who had moderate to severe vomiting symptoms were 2.26 times more likely to suffer from prenatal depression compared to those who had no or minimal vomiting symptoms. Considering the bi-directional mechanisms between gastrointestinal and psychological symptoms, it is reasonable to accept such an association (19).

This study suggested that every increased point in the PSRS would increase the risk of prenatal depression by 5.48 times, defining prenatal stress as a predominant contributor to the condition. The close association between stress and depression has been well-documented, not only in pregnant women but also in the general population (48, 49). Stress could have profound effects on the development of depression either in a direct pathway via epigenetic changes or in an indirect pathway mediated by multiple variables, for example, anxiety symptoms (48–50).

### The novel nomograms were valid for the risk prediction of prenatal depression

A variety of methods and parameters were used to assess the validity and performance of the novelly constructed nomograms for the risk prediction of prenatal depression. Several parameters suggested model II is superior considering its better performance in discriminative efficiency, predictive efficiency, and clinical utility. Moreover, it could be used for the precise risk estimation of individuals with extremely high risk of prenatal depression (0.6−0.95, as is shown in **Figure 1**). However, it does not necessarily indicate that model I is not valid or meaningful. The model I reached a satisfactory level in all the tested parameters. Besides, it could be advantageous when used as a quick screening tool as healthcare professionals will not spend time on stress assessment, which could be time-consuming in the busy clinical setting.

After a comprehensive search of relevant evidence, only one recently proposed nomogram for the risk prediction of prenatal depression was identified (23). The referenced model included nine input variables, namely employment, planned pregnancy, pregnancy number, conception methods, gestational diabetes mellitus, twin pregnancy, placenta previa, umbilical cord encirclement, and educational attainment. Despite its large sample size and equivalent discriminative efficiency compared to the current study, it only included pregnant women in the third trimester, which could limit its generalizability. Moreover, some other important candidate variables were neglected in the risk prediction model construction, such as relationships with family members and the history of gynecologic diseases.

### Strengths and limitations

This study has several remarkable strengths. Foremostly, it is among the very limited studies that developed monograms for the precise individualized risk prediction of prenatal depression, which contributed innovative evidence to the knowledge body. Moreover, several previously underestimated factors were examined for the association with prenatal depression. Besides, multiple parameters were employed to assess the performance of the nomograms, the agreed positive results improved the validity of the risk prediction models.

Despite its strengths, the findings of this study should be interpreted with the consideration of the following limitations. First, the research participants were recruited from two research sites with a convenience sampling approach, which could introduce selection bias to the study. Second, formal sample size estimation was not performed in this study, and therefore, there is the risk that the sample size is not adequate enough. Third, participants in the training and validation sets were from the same population, making the external validity of the risk prediction models uncertain. Fourth, a large proportion of the variables were measured in a subjective manner, which might lead to reporting bias. Besides, due to the nature of a cross-sectional design, the causal relationship between the associated factors and prenatal depression was not guaranteed.

### Implications

In view of the relatively high prevalence of prenatal depression among pregnant women and its burdensome negative consequences, regular assessment and early preventive strategies should be included in the routine care of obstetrics clinics. The nomograms constructed in the current study could be made use of for the early detection of pregnant women highly vulnerable to prenatal depression, ideally in an auto-generated approach making use of the health data in the hospital information systems.

To examine the external validity of the novelly constructed nomograms for the risk prediction of prenatal depression, large-scale, multi-centered studies originating from diverse populations are necessary. Due to the very limited evidence regarding the association between the antecedent history of gynecologic diseases and prenatal depression, and the inconsistent findings on the association between trimester and prenatal depression, further studies examining such associations are valuable. In addition, longitudinal studies are desirable to confirm the causal relationship between the candidate risk factors and prenatal depression.

## Conclusions

The prevalence of prenatal depression was considerably high in pregnant women. Risk factors associated with prenatal depression included unemployment, poor relationship with partners, antecedent history of gynecologic diseases, unplanned pregnancy, an earlier stage of pregnancy, more severe vomiting symptoms, and prenatal stress. Both of the two novelly constructed nomograms for the risk prediction of prenatal depression had good discriminative and predictive performance and clinical utility. The risk prediction model I could be feasible for fasting screening, while model II could generate more precise risk estimations.

## Data Availability

The raw data supporting the conclusions of this article will be made available by the authors, without undue reservation.
