# Supplementary figures and images for: The construction and validation of the novel nomograms for the risk prediction of prenatal depression: a cross-sectional study
Source: Front Psychiatry. 2024 Nov 29;15:1478565. doi: 10.3389/fpsyt.2024.1478565 (PMC11640862; doi:10.3389/fpsyt.2024.1478565)

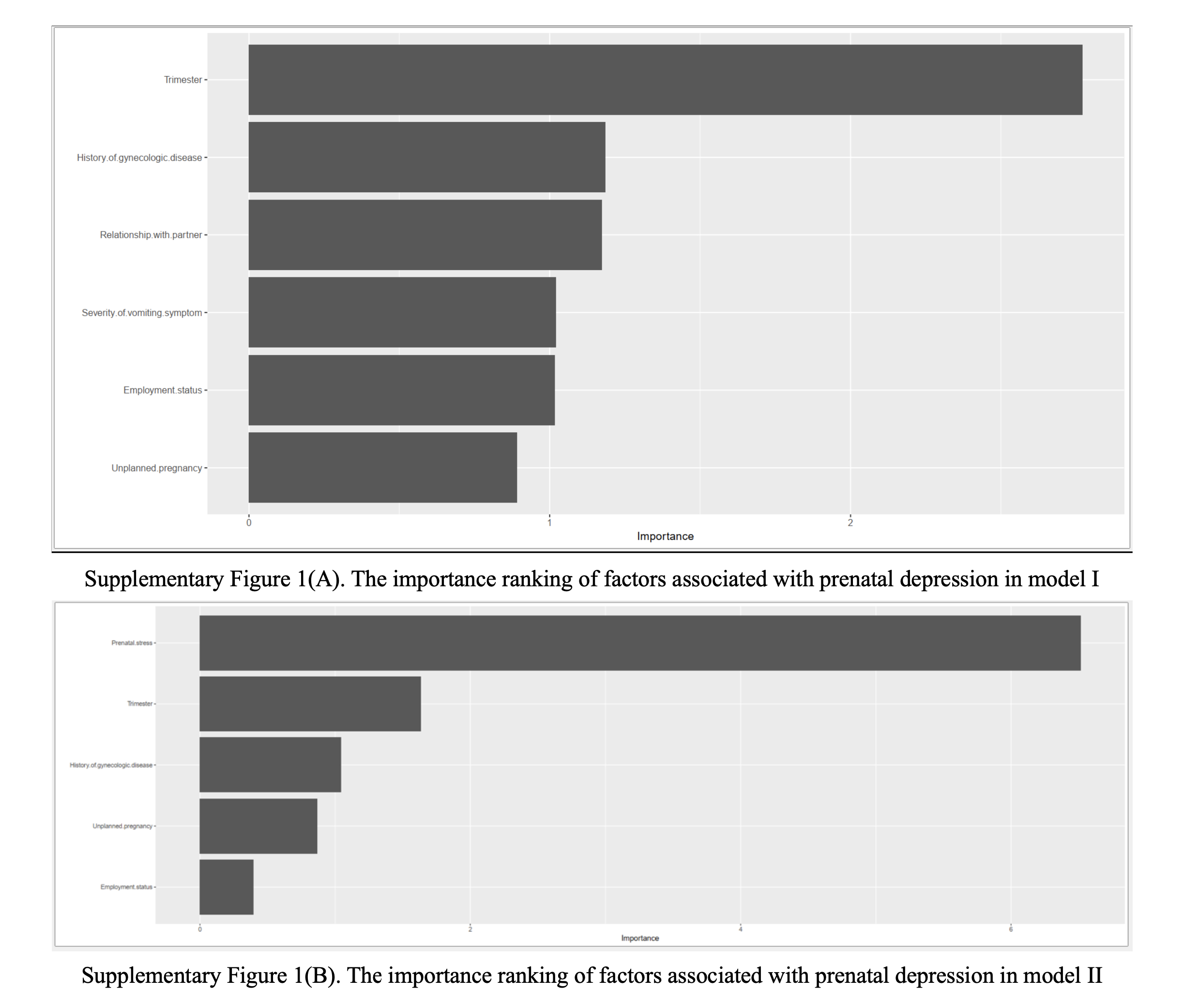

Supplement: Supplementary file 1 [file Image1.jpeg]
